# Effects of Exogenous Selenium on Accumulations of Selenium, GABA and Antioxidant Activity of Chestnut During Germination

**DOI:** 10.3390/molecules31111847

**Published:** 2026-05-27

**Authors:** Haifen Wang, Weiwei Liu, Fei Peng, Ziye Zhang, Jiawei Cao, Jiayu Shi, Liang He, Yunbin Jiang, Mengshi Wang, Junwei Yuan

**Affiliations:** 1 Research Center of Rural Vitalization, Hebei Normal University of Science and Technology, Qinhuangdao 066004, China; haifenwang81@126.com (H.W.); feipeng1986@hotmail.com (F.P.); 2 Chestnut Research Center, Hebei Normal University of Science and Technology, Qinhuangdao 066004, China; weiweiliu08@163.com (W.L.); zzziye@126.com (Z.Z.); m17864497526@163.com (J.C.); sjiayuup@163.com (J.S.); lianghe825@163.com (L.H.); yunbinjiang@126.com (Y.J.); wms15233551083@163.com (M.W.); 3 College of Food Science and Technology, Hebei Normal University of Science and Technology, Qinhuangdao 066004, China

**Keywords:** chestnut, germination, selenium uptake, γ-aminobutyric acid, antioxidant activity

## Abstract

The objective of this study was to investigate the effect of exogenous selenium on selenium enrichment and antioxidant activity of germinated chestnuts. We treated ‘Zaofeng’ chestnuts with Na_2_SeO_3_ at concentration of 0, 20, 40, 60 and 80 mg/L, and analyzed, during germination, the level of total Se, SeCys_2_, MeSecys, Se^IV^, SeMet, Se^VI^, γ-aminobutyric acid (GABA), antioxidant enzyme (phenylalanine ammonialyase (PAL), glutathione peroxidase (GPX), superoxide dismutase (SOD) and catalase (CAT)) activity, non-enzymatic antioxidant substances (total polyphenols and flavonoids) content and antioxidant capacity (DPPH, ABTS). The results indicated that low concentrations of selenium (20–40 mg/L) significantly promoted the organic transformation of selenium, with a Se-enrichment rate over 74%. Antioxidant enzyme (PAL, SOD, CAT) activities and total phenol content were enhanced by 1.1 to 1.9-fold compared with the control, leading to a 12.2–29.2% improvement in antioxidant capacity (DPPH and ABTS). In contrast, the high concentration of selenium (80 mg/L) induced oxidative stress, inhibiting enzyme (PAL, SOD, CAT) activities (reduced by 14.1–20.5%) and decreasing antioxidant capacity (DPPH) by approximately 19.0%. During chestnut germination, selenite was absorbed by the embryo and subsequently transformed into organic Se in vivo, ultimately being stored mainly as SeCys_2_. The selenium enrichment rate decreased significantly with increasing Na_2_SeO_3_ treatment concentration: from 86.4% at 20 mg/L to 62.2% at 80 mg/L. Furthermore, treatment with 40 mg/L Na_2_SeO_3_ led to a significant increase in GABA content of germinated chestnuts, reaching 1.3 times that of the control group. Overall, germination with 20–40 mg/L Na_2_SeO_3_ is an effective condition for producing Se-enriched chestnut sprouts with enhanced GABA and antioxidant capacity, offering a potential functional food ingredient.

## 1. Introduction

Selenium (Se) is an essential trace element for human health, playing a vital role in various biological functions such as anti-cancer, antioxidation, anti-aging, heart protection, vision protection, immunity improvement, and reproductive capacity enhancement [1,2]. Prolonged Se deficiency can result in sub-health and predispose individuals to a range of associated ailments [3,4]. However, due to the variation in soil Se levels across different regions, the Se content in food sources, particularly organic Se, is generally low in many areas of the world [5]. One viable approach for Se supplementation is to enhance the Se concentration in agricultural products through exogenous Se biofortification [6]. Among various biofortification approaches, seed germination offers a short-term, low-cost strategy to increase Se bioavailability [7]. Assessing the conversion efficiency of organic Se in agricultural products and ensuring balanced nutrition are crucial for the safe cultivation of Se-enriched foods.

Common inorganic forms include SeO^2−^_4_ and SeO^2−^_3_, while major organic forms include SeCys_2_, MeSeCys, and SeMet [8,9]. While inorganic Se is abundant and cost-effective, organic Se is primarily derived from the conversion of inorganic forms within organisms [10]. Currently, the biological enrichment of exogenous Se using plant seeds as raw materials is recognized as the convenient, efficient, and safe technology for Se-enriched agricultural production [11,12,13]. Seed sprouts are known to accumulate Se from various inorganic sources [14,15] and incorporate Se into newly synthesized proteins during sprout growth [16]. Studies have shown that bean sprouts germinated under inorganic Se treatment for 5 to 7 days exhibited significantly higher Se content compared to untreated bean sprouts [10]. Inorganic Se treatment has also been found to enhance Se absorption by garden cress sprouts [17]. During germination, Se accumulates in brown rice, primarily in Se-containing proteins [18]. Studies on various Se-enriched foods, including mushroom [19], ovalbumin [20], and *Zea mays* [21], have confirmed the potent effects of Se on specific intracellular selenoproteins and crucial antioxidant activities. Furthermore, the germination of seeds has been shown to increase the content and activity of bioactive compounds including polyphenols, flavonoids, vitamins, and free amino acids, particularly γ-aminobutyric acid (GABA) [22]. GABA, an essential inhibitory neurotransmitter in the central nervous system, plays a key role in enhancing sleep quality and reducing blood pressure [23]. With favorable water solubility, thermal stability, and safety for consumption, GABA finds wide application in food production [24].

In recent years, researchers have focused on optimizing the technological parameters for Se and GABA enrichment during crop germination [25]. Although Se is known to modulate stress-induced GABA accumulation in some plants, no information is available on whether exogenous Se affects GABA metabolism in germinated chestnut. In our previous study, the GABA content and antioxidant capacity of chestnuts were significantly increased by the germination process [26]. Several studies have demonstrated Se biofortification in legume sprouts, but similar work in tree nuts such as chestnuts is lacking, and the effects of Se-biofortification on the secondary metabolites of germinated chestnuts remain unclear.

In this study, Se-enriched chestnuts were cultivated under varying concentrations of Na_2_SeO_3_ solution to evaluate the accumulation of selenium in different parts of germinated chestnuts, and the changes of the antioxidant enzyme activity system. Meanwhile, the GABA, total polyphenol, flavonoid content and antioxidant capacity were assessed. This study aimed to (1) determine the optimal Se concentration for chestnut germination, (2) quantify the accumulation of Se and GABA, and (3) evaluate the changes in antioxidant activity under Se treatment. This present work focuses on nutritional biofortification rather than stress-tolerance studies, and this knowledge gap is practically relevant for developing Se-enriched sprouted nut products.

## 2. Results and Discussion

### 2.1. Effects of Exogenous Na_2_SeO_3_ on Total Se Content in Germinated Chestnuts

**Figure 1:**
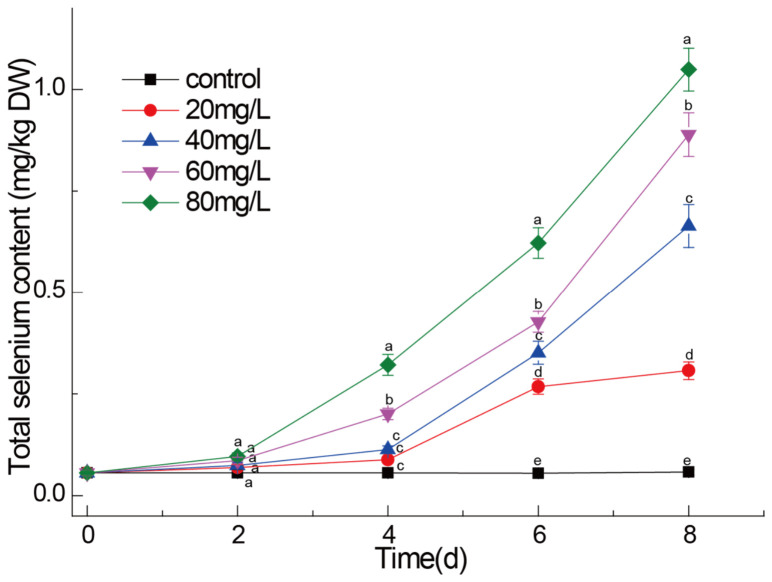
Dynamic changes of total selenium content in germinated chestnuts under different concentrations of Na_2_SeO_3_ treatments. Note: Values in the figure were shown as the means ± standard error (*n* = 3). Vertical bars represent the standard errors of the means. Different letters represent significant differences under different Na_2_SeO_3_ treatments, *p*-value < 0.05.

With the increase in exogenous Na_2_SeO_3_ concentration and the prolongation of germination time, the total selenium content in germinated chestnuts increased significantly (*p* < 0.05) (Figure 1). In the control, the total Se content in chestnuts remained within the range of 0.056 to 0.058 mg/kg throughout the germination process, with no significant differences observed (*p* > 0.05). The selenium of chestnuts under four concentrations of exogenous Na_2_SeO_3_ did not increase significantly in the first 2 days (*p* > 0.05). This may be because the sodium selenite on the surface of the chestnuts was not absorbed into chestnut tissue 2 d after sowing, and was washed away during germination to prepare samples. Under 80 and 60 mg/L Na_2_SeO_3_ treatment, the total Se content in chestnuts significantly increased (*p* < 0.05) from the 4th day, reached 1.049 and 0.889 mg/kg by the 8th day post-sowing respectively. Under 40 and 20 mg/L Na_2_SeO_3_ treatment, the total Se content in chestnuts significantly increased (*p* < 0.05) from the 6th day. The applied 20, 40, 60 and 80 mg/L Na_2_SeO_3_ correspond to actual selenium concentrations of approximately 9.1, 18.2, 27.4 and 36.5 mg Se/L, respectively. These results indicated that germination in the presence of a Na_2_SeO_3_ solution is an efficient method for Se biofortification of germinated chestnuts.

In this study, the control chestnuts sourced from the primary chestnut-producing region in Qianxi county, Tangshan city, Hebei province, China, exhibited a total Se content ranging from 0.056 to 0.058 mg/kg, significantly below the recommended standard for Se-enriched food by China Nutrition Society (0.1 to 1.0 mg/kg) [27]. Different plant species exhibit varying capacities for Se uptake and accumulation. For instance, rye grains treated with selenite accumulated Se up to 55 µg/g during germination [28], while alfalfa germination enriched Se concentrations up to 150 µg/g DW [29]. The Se content accumulated by garden cress germinated with 8 mg/L selenite solution (39 μg/g) surpasses that of lupine bean sprouts grown under the same conditions (11 μg/g dm) [17]. Therefore, the appropriate Se treatment and germination duration are pivotal factors in producing high-quality Se-enriched sprouts. Germinated chestnuts treated with 20 and 40 mg/L for 6–8 days, 60 mg/L for 4–8 days, 80 mg/L Na_2_SeO_3_ for 4–6 days all could serve as viable sources for Se-enriched foods, meeting the regulatory standards for Se enrichment in foods and agricultural products [6]. Se accumulation during different germination stages involves distinct processes. In the initial stage of germination (2–4 d after sowing), seeds absorb selenite and water from the environment, leading to swelling and restoration of metabolic activity [30]. During this period, selenite enters the seeds and cells through the aleurone layer with the absorbed water [18]. The total Se content in the germ experiences a rapid increase during the middle–late stage (4–8 d after sowing) of germination, a pattern similar to observations in mung beans germinated via Na_2_SeO_3_ treatment [10].

### 2.2. Effects of Exogenous Na_2_SeO_3_ on Se Speciation and Content in Germinated Chestnut

**Figure 2:**
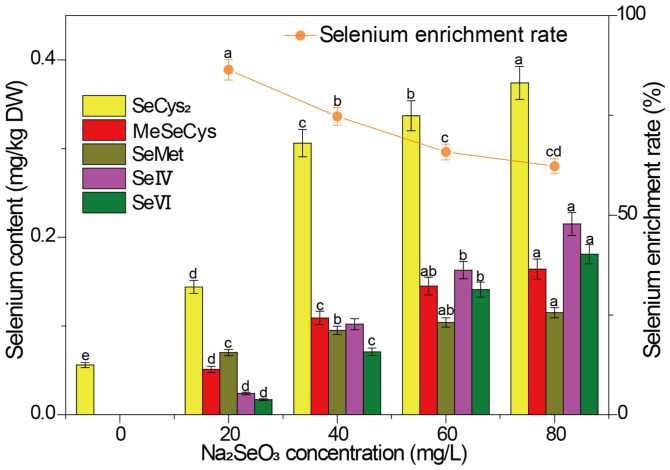
The effects of different concentrations of Na_2_SeO_3_ treatments on SeCys_2_, MeSeCys, SeMet, Se^IV^, Se^VI^ and the selenium enrichment rate in germinated chestnuts. Note: Values in the figure were shown as the means ± standard error (*n* = 3). Vertical bars represent the standard errors of the means. Different letters represent significant differences under different Na_2_SeO_3_ treatments, *p*-value < 0.05.

Due to the low bioavailability and high toxicity of inorganic Se [31], efforts should be directed towards enhancing the organic speciation of Se [32]. Considering nutritional value and safety aspects, the organic Se content holds greater significance, underscoring the importance of understanding Se form and transformation during plant germination. The Na_2_SeO_3_ treatment resulted in a significant (*p* < 0.05) increase in SeCys_2_, MeSecys, SeMet, Se^IV^ and Se^VI^ content of germinated chestnuts, while Se-enrichment rate decreased gradually (Figure 2). On the 8th day after sowing, the SeCys_2_ content ranged from 0.056 to 0.374 mg/kg, the MeSeCys content ranged from 0 to 0.164 mg/kg, the SeMet content ranged from 0 to 0.115 mg/kg, and the Se-enrichment rate reduced from 86.42% to 62.24%. After treatment with sodium selenite, selenium in germinated chestnuts mainly exists in organic form, which indicates that germinated chestnuts can effectively transform and accumulate organic selenium. Similar results also appeared in the experiment of mung bean germination treated by Na_2_SeO_3_ soaking [33]. Plants primarily convert inorganic Se into selenomethionine and incorporate it into protein [34]. Se-Met is the main form of organic selenium in cereals and leguminous plants, while the main selenium compound in selenium-enriched plants such as garlic, onion and wild leek is SeMeCys [35]. The main forms of organic selenium in different parts of some selenium-enriched plants are also significantly different. For example, in selenium-enriched broccoli, the major form of selenium in roots is Se-Met, while the main form of selenium in fruits is SeMeCys [36]. In this study, SeCys_2_ was the most abundant and stable form of Se detected in chestnuts under the experimental conditions used. After being absorbed by plants, inorganic selenium will be mixed with Cys and Met to form Se-Cys and Se-Met respectively, and then further integrated into protein to form selenoprotein [17]. When cruciferous plants are cultivated in Se-rich soil, they convert less than 40% of the total Se content into selenoamino acids [37]. In contrast, when grown in Se-poor conditions, primary species can incorporate over 95% of the total Se content as selenoamino acids [38,39]. In this study, the Se-enrichment rate decreased gradually with the increase in Na_2_SeO_3_ concentration in this study. This may be because when the concentration of Na_2_SeO_3_ reaches a certain threshold, the ability of Se assimilation and transformation into organic selenium decreases. This is basically consistent with the research results of selenium-enriched soybean sprouts [40].

### 2.3. Effects of Exogenous Na_2_SeO_3_ on Phenylalanine Ammonialyase (PAL), Glutathione Peroxidase (GPX), Superoxide Dismutase (SOD) and Catalase (CAT) Activity of Germinated Chestnuts

**Figure 3:**
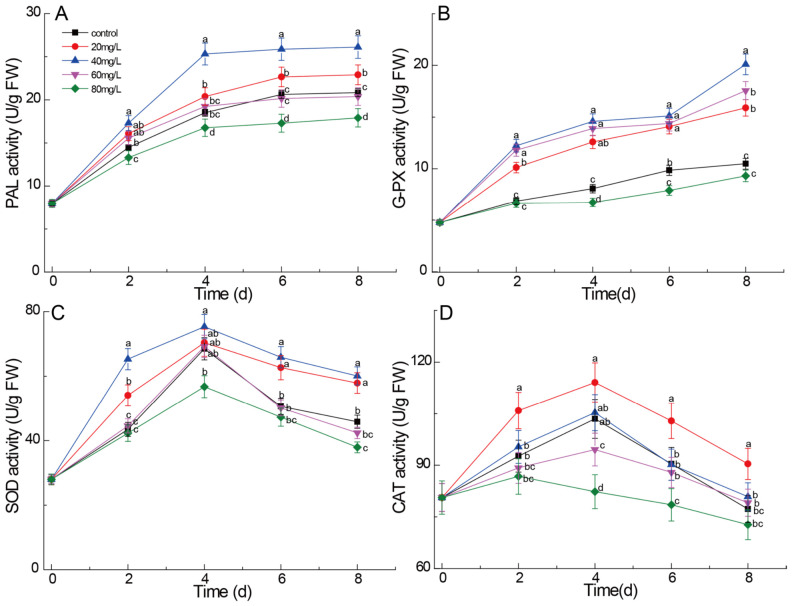
Dynamic changes of PAL (**A**), GPX (**B**), SOD (**C**) and CAT (**D**) of germinated chestnuts under different concentrations of Na_2_SeO_3_ treatments. Note: Values in the figures were shown as the means ± standard error (*n* = 3). Vertical bars represent the standard errors of the means. Different letters represent significant differences under different Na_2_SeO_3_ treatments, *p*-value < 0.05.

PAL is a key enzyme in the phenylpropane metabolic pathway [41]. As shown in Figure 3A, PAL activity first increased (0–4 d after sowing) and then stabilized (4–8 d after sowing) during germination. Low concentrations of Na_2_SeO_3_ (20 and 40 mg/L) significantly enhanced PAL activity during the whole germination period (*p* < 0.05). It is worth noting that the PAL activity reached the highest at 40 mg/L, reaching 26.11 U/g FW on the 8th day after sowing, which was 25.2% higher than that of the control group. This finding is consistent with previous research reports, indicating that sodium selenite can enhance PAL activity in many plant systems. For example, the PAL activity of chicory was increased by 36.4% by the combined treatment of NO and nano-Se, while the PAL activity of bitter gourd seedlings was increased by 39% by nano-Se [42]. In contrast, high concentration of Na_2_SeO_3_ (80 mg/L) inhibited PAL activity. On the 8th day after sowing, it decreased to 17.92 U/g FW, which was 14.1% lower than that of the control group. This inhibition may be attributed to the excessive accumulation of reactive oxygen species exceeding the antioxidant capacity of cells, leading to oxidative damage of enzyme proteins and destruction of redox homeostasis of cells [43]. There was no significant difference in PAL activity between 60 mg/L treatment and control group (*p* > 0.05).

In all selenium treatment groups, GPX activity increased continuously during the whole germination period, and reached the maximum on the 8th day (Figure 3B). GPX activity in the control group increased moderately from 4.77 U/g FW on day 0 to 10.48 U/g FW on day 8. GPX, as a selenase containing selenium in the form of selenocysteine in its active center, is the most sensitive to selenium treatment among all the detected enzymes. The activity of GPX increased significantly under the treatment of 40 mg/L selenium (1.92 times that of the control group), which highlighted the efficiency of selenium bioaugmentation in germinated chestnuts. The 20 and 60 mg/L treatments also significantly increased GPX activity, reaching 15.90 and 17.56 U/g FW respectively. This result confirmed earlier studies, and showed that trace selenium could enhance GPX activity in germinated seeds of wheat, rice and various vegetables [44]. However, the 80 mg/L treatment reduced GPX activity to 9.31 U/g FW on the 8th day, which was only slightly lower than that of the control group (*p* > 0.05), indicating that it was inhibited under excessive selenium concentration. This shows that excessive selenium interferes with the synthesis or stability of selenase [45]. This inhibition may be due to selenium-induced oxidative stress interfering with protein biosynthesis [46].

SOD serves as the first line of defense against superoxide anions, catalyzing the dismutation of O_2_^−^· into H_2_O_2_ and O_2_ [47]. Unlike PAL and GPX, SOD activity rapidly increased from day 0 to day 4 across all groups, peaked on day 4, and then gradually declined by day 8 (Figure 3C), consistent with the typical unimodal curve of SOD during seed germination. At the peak (day 4), SOD activity in the control group was 68.51 U/g FW. Treatment with 40 mg/L Se significantly increased SOD activity to 75.32 U/g FW, which was 9.9% higher than the control. The 40 mg/L treatment maintained the highest SOD activity throughout germination. In contrast, the 80 mg/L treatment resulted in SOD activity of 56.76 U/g FW on day 4, 17.1% lower than the control. The promoting effect of low Se concentration (20–40 mg/L) on SOD activity is consistent with previous findings in alfalfa seeds [29]. A possible explanation for this activation is that Se at low concentrations may induce mild oxidative stress, which could in turn upregulate the expression of antioxidant enzyme-encoding genes (including SOD) via ROS-mediated signaling pathways involving transcription factors such as WRKY and DREB, as suggested by previous studies [48]. However, direct evidence for this mechanism in germinating chestnuts is still lacking.

With the prolongation of germination time, CAT activity in all treatment groups showed a trend of first increasing and then decreasing, peaking on day 4 (Figure 3D). The effect of different selenium concentrations on CAT activity was concentration-dependent: low-concentration (20 mg/L) treatment significantly increased CAT activity at all time points (*p* < 0.05), reaching a maximum of 114.09 U/g FW on day 4, which was 10.2% higher than the control. The 40 mg/L Na_2_SeO_3_ treatment resulted in slightly higher CAT activity than CK, but the difference was not significant (*p* > 0.05). The 60 and 80 mg/L Na_2_SeO_3_ treatments significantly inhibited CAT activity, with the 80 mg/L Na_2_SeO_3_ showing a 20.5% reduction compared to the control on day 4, and remaining at the lowest level throughout germination. Low-concentration Se significantly increased CAT activity during chestnut germination, consistent with the antioxidant effects of selenium in other plants [49,50]. When Se concentration reached 60 mg/L or higher, CAT activity was markedly inhibited. High Se concentration causes excessive selenium accumulation in plants, inducing bursts of reactive oxygen species (ROS) such as superoxide anion (O_2_^−^) and hydrogen peroxide (H_2_O_2_). Additionally, selenium binds to sulfhydryl (-SH) groups, disrupting the structure of enzyme proteins and inactivating the CAT active center [51]. Of note, although CAT activity was inhibited under high Se treatment, a small increase followed by a rapid decline was still observed in the early germination stage (0–2 d after sowing), indicating that seeds can still initiate a limited antioxidant response at the beginning of germination, but this response is quickly suppressed as selenium toxicity intensifies.

The decrease in SOD and CAT activities after day 4 of germination (Figure 3C,D) can be attributed to several factors. First, the early stage of germination (days 0–4) involves intense metabolic reactivation and ROS production, which induces antioxidant enzymes. After day 4, the stored reserves are partially depleted, and the overall metabolic rate may slow down, leading to reduced enzyme synthesis. Second, in the presence of exogenous selenium, a hormetic effect may occur: low Se transiently stimulates antioxidant enzymes, but prolonged exposure or higher Se levels could cause moderate oxidative damage, suppressing enzyme activity. Third, it is known that in many seeds, the peak of antioxidant enzyme activity coincides with radicle emergence, after which activity naturally declines as the seed shifts from germination to early growth. Future studies measuring enzyme protein levels and gene expression could further clarify the regulation.

In a word, Na_2_SeO_3_ synergistically regulates the antioxidant enzyme network in germinating chestnuts. At 20–40 mg/L, selenium enhances the activities of PAL, SOD, GPX, and CAT, thereby strengthening H_2_O_2_ scavenging and phenolic biosynthesis pathways, whereas a high concentration (80 mg/L) causes irreversible oxidative damage.

### 2.4. Effects of Exogenous Na_2_SeO_3_ on GABA Content in Germinated Chestnuts

**Figure 4:**
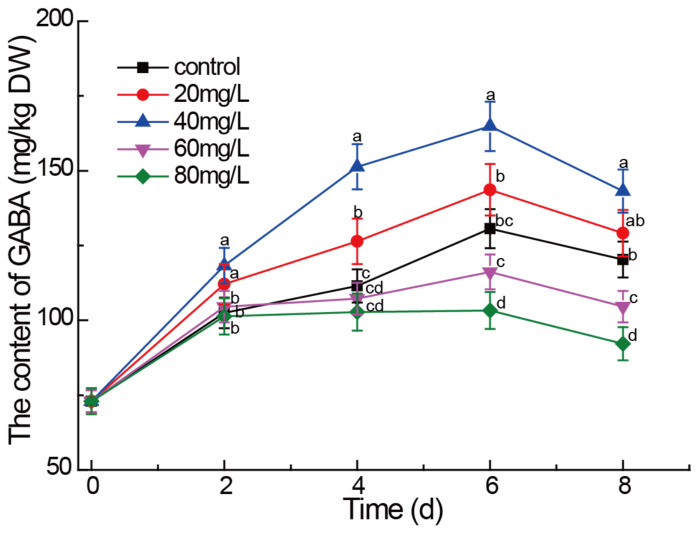
Comparison of γ-aminobutyric acid (GABA) content in chestnut under different concentrations of Na_2_SeO_3_ treatments. Note: Values in the figure were shown as the means ± standard error (*n* = 3). Vertical bars represent the standard errors of the means. Different letters represent significant differences under different Na_2_SeO_3_ treatments, *p*-value < 0.05.

The changes in GABA content during chestnut germination under different Na_2_SeO_3_ treatments are shown in Figure 4. In all treatment groups, GABA content gradually increased from day 0 to day 6, reflecting the natural activation of GABA biosynthesis during germination, peaked at day 6, and then decreased from day 6 to day 8. The 40 mg/L Na_2_SeO_3_ treatment consistently maintained the highest GABA content throughout germination, reaching 164.87 mg/kg DW on day 6, which was 26.2% higher than the control (*p* < 0.05). In contrast, the 80 mg/L treatment resulted in GABA levels 20.9% lower than the control (*p* < 0.05), indicating that the inhibitory effect of high Se concentration becomes more pronounced during the late germination stage when GAD activity is naturally elevated. Low Se concentrations (20 and 40 mg/L) significantly promoted GABA accumulation throughout the germination period, whereas the high Se concentration (80 mg/L) inhibited GABA accumulation.

The stimulatory effect at low Se concentrations (20–40 mg/L) can be attributed to the induction of mild oxidative stress. Sub-toxic levels of selenium generate moderate amounts of reactive oxygen species (ROS), which act as signaling molecules to activate stress-responsive transcription factors and upregulate the expression of GAD and other genes involved in GABA metabolism [52]. This interpretation is consistent with the recognition of GABA as a novel protective agent capable of mitigating oxidative stress [53]. In a study of cut lily flowers, exogenous GABA treatment elevated antioxidant enzyme activities, prevented hydrogen peroxide accumulation, and maintained higher levels of total phenols and soluble proteins, thereby delaying senescence [54]. Conversely, the increase in endogenous GABA accumulation under mild selenium-induced stress may similarly contribute to the antioxidant defense system of germinating chestnuts. At high Se concentration (80 mg/L), the capacity of the cellular antioxidant system is overwhelmed, leading to excessive ROS accumulation, which causes oxidative damage to the GAD enzyme protein, disruption of cellular redox homeostasis, and inhibition of GABA biosynthesis [55]. The reduction in GABA content under the 80 mg/L Se treatment (20.9% lower than the control) is largely consistent with results observed in highland barley seeds [56].

The optimal Se concentration for GABA enhancement identified in this study (40 mg/L Na_2_SeO_3_) differs from the concentrations reported for other crop species. In foxtail millet, the appropriate concentrations were 60 mg/L for soaking and 2 mg/L for spraying [25]. These differences may reflect species-specific variations in Se sensitivity, as well as differences in experimental conditions including germination duration, temperature, seed size, and Se application method. The chestnut, as a large-seeded species with substantial nutrient reserves, may exhibit distinct patterns of GABA accumulation and Se responsiveness compared to small-seeded cereals or legumes. The observation that GABA is the major amino compound during chestnut germination and early seedling growth highlights the particular importance of this metabolite in the chestnut’s nitrogen metabolism and stress adaptation.

### 2.5. Effects of Exogenous Na_2_SeO_3_ on Total Polyphenols and Flavonoids Content in Germinated Chestnuts

**Figure 5:**
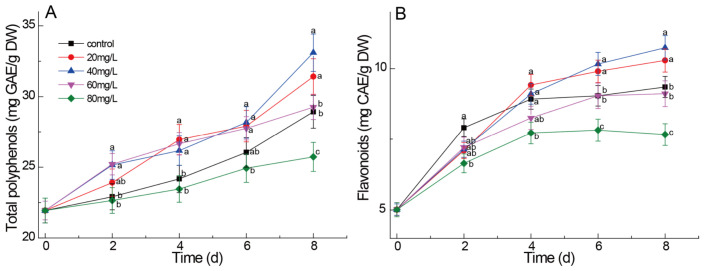
Dynamic changes of total polyphenol (**A**) and flavonoid (**B**) content of germinated chestnut under different concentrations of Na_2_SeO_3_ treatments. Note: Values in the figures were shown as the means ± standard error (*n* = 3). Vertical bars represent the standard errors of the means. Different letters represent significant differences under different Na_2_SeO_3_ treatments, *p*-value < 0.05.

Under all treatments, the total polyphenols content (TPC) in germinating chestnuts gradually increased throughout the 8-day germination period (Figure 5A), reflecting the natural activation of phenylpropanoid metabolism during seed germination. On day 8, the TPC in the control group (0 mg/L) was 28.92 mg GAE/g DW. Low selenium concentrations (20 and 40 mg/L) significantly increased TPC, with the highest value (33.11 mg GAE/g DW) observed at 40 mg/L, which was 14.5% higher than the control. The TPC at 20 mg/L (31.42 mg GAE/g DW) was also slightly higher than the control, but the difference was not significant (*p* > 0.05). These findings are generally consistent with observations in other germinated seeds [57]. In contrast, the 80 mg/L Na_2_SeO_3_ treatment reduced TPC to 25.72 mg GAE/g DW, which was 11.1% lower than the control, indicating that phenolic biosynthesis is inhibited under excessive selenium stress.

Across all treatments, total flavonoid content (TFC) in germinating chestnuts accumulated rapidly during the first 4 days, and the accumulation rate slowed down during the later period (4–8 days) (Figure 5B). Low selenium concentrations (20 and 40 mg/L) showed no significant difference in TFC from the control during the first 4 days (*p* > 0.05), but TFC was significantly higher than the control from day 6 to day 8 (*p* < 0.05). These findings are generally consistent with increased flavonoid accumulation observed in other selenium-biofortified germinated seeds [58,59]. In contrast, the 80 mg/L treatment reduced TFC to 7.66 mg CAE/g DW, which was 18.1% lower than the control.

Low concentrations of Na_2_SeO_3_ (20–40 mg/L) significantly promoted the accumulation of total polyphenols and flavonoids in germinated chestnuts, indicating that appropriate selenium biofortification during seed germination can effectively enhance the accumulation of phenolic compounds in various crops. For example, in black soybeans [57], selenium biofortification significantly increased total phenolic and total flavonoid contents in germinated seeds. Similarly, selenium-enriched germination significantly increased total phenolic and total flavonol contents in hulless barley seeds [56] and soybeans [40]. It is speculated that the underlying mechanism is the activation of the phenylpropanoid pathway in germinating chestnuts, which is generally consistent with the changes in PAL activity described above (Figure 3A).

### 2.6. Effects of Exogenous Na_2_SeO_3_ on Antioxidant Capacity of Germinated Chestnuts

**Figure 6:**
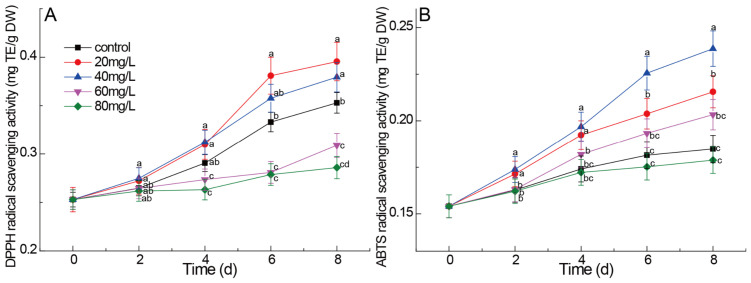
Dynamic changes of DPPH (**A**) and ABTS (**B**) radical scavenging activity of germinated chestnuts under different concentrations of Na_2_SeO_3_ treatments. Note: Values in the figures were shown as the means ± standard error (*n* = 3). Vertical bars represent the standard errors of the means. Different letters represent significant differences under different Na_2_SeO_3_ treatments, *p*-value < 0.05.

DPPH radical scavenging activity, a widely used indicator of antioxidant capacity based on hydrogen atom transfer, showed a gradual increase throughout the germination period across all treatments (Figure 6A). This reflected the natural accumulation of phenolic compounds and other antioxidants during seed germination, consistent with the enhanced PAL activity and increased phenolic content observed in this study. On day 8, the DPPH scavenging activity in the control (0 mg/L) was 0.353 mg TE/g DW. Low selenium concentrations (20 mg/L) significantly increased DPPH scavenging activity, which was 12.2% higher than the control. These results are generally consistent with findings in foxtail millet sprouts [25]. In contrast, the effect of high selenium concentration on DPPH scavenging activity gradually weakened. The DPPH scavenging activity at 80 mg/L on day 8 was 0.286 mg TE/g DW, which was 19.0% lower than the control, indicating that hydrogen atom transfer-based antioxidant capacity is inhibited under excessive selenium stress.

ABTS radical scavenging activity reflects antioxidant capacity based on electron transfer. Similar to DPPH scavenging activity, ABTS radical scavenging activity increased steadily throughout the germination period, reaching a maximum on day 8 across all treatments (Figure 6B). The ABTS scavenging activity in the control group on day 8 was 0.185 mg TE/g DW. Low selenium concentrations significantly increased ABTS scavenging activity, with the highest value (0.239 mg TE/g DW) observed at 40 mg/L, which was 29.2% higher than the control. The 20 mg/L treatment also significantly increased ABTS scavenging activity to 0.216 mg TE/g DW (16.5% higher than the control). These findings are consistent with increased ABTS radical scavenging activity observed in germinated seeds of mung bean [60]. The ABTS scavenging activity of germinated chestnuts under 80 mg/L treatment was 0.179 mg TE/g DW (slightly lower than the control), but the difference was not significant (*p* > 0.05).

Notably, ABTS scavenging activity was consistently higher than DPPH scavenging activity across all treatments. This observation is not uncommon, as ABTS and DPPH radicals differ in their redox potential, solubility, and reaction kinetics, and the two assays often yield different absolute values when applied to the same sample matrix. This study demonstrates that low concentrations of Na_2_SeO_3_ (20–40 mg/L) significantly enhance both DPPH and ABTS radical scavenging activities in germinating chestnuts, indicating that selenium biofortification during seed germination can effectively improve the total antioxidant capacity of various crops. In foxtail millet sprouts, soaking with 60 mg/L Na_2_SeO_3_ combined with spraying 2 mg/L Na_2_SeO_3_ significantly increased free and bound phenolic contents, thereby enhancing in vitro antioxidant activity [25]. Similarly, mung bean seeds and sprouts treated with 30 mg/L Na_2_SeO_3_ exhibited significantly elevated ABTS and DPPH radical scavenging activities, accompanied by specific enrichment of benzoic acid, rutin, and luteolin glycoside [61]. Wheat sprouts also showed increased ABTS, DPPH, and SOD activities under moderate selenium biofortification (0.25–0.50 mg/L) [62]. Peanut seeds soaked with 7.5 μmol/L Na_2_SeO_3_ exhibited significantly improved antioxidant capacity and sprout quality [63]. Furthermore, chickpea sprouts treated with 2 mg/100 g seeds Na_2_SeO_3_ showed a significant 33% increase in antioxidant capacity [64].

Overall, the enhancement of antioxidant properties by Se treatment can be explained by the ‘stress-response’ hypothesis [44]. Selenium at low (20 mg/L) to moderate (40 mg/L) concentrations generates reactive oxygen species (ROS) at sub-toxic levels, which in turn upregulates the expression and activity of antioxidant enzymes as a defense mechanism [51]. This is consistent with the well-known hormetic effect of Se in plants. In this study, the highest Se concentration (80 mg/L) caused a decrease in antioxidant enzyme activities, indicating that the stress exceeded the adaptive capacity of the chestnut tissue. Therefore, the observed increase in antioxidant activity is not due to a direct radical-scavenging role of Se itself, but rather to Se-induced mild oxidative stress that triggers an endogenous antioxidant response.

### 2.7. Practical Implications and Limitations

Based on our laboratory-scale results, germination with 20 mg/L sodium selenite is effective for producing Se-enriched chestnut sprouts with enhanced GABA and antioxidant capacity. However, direct extrapolation to open-field or commercial farming requires caution. In a controlled germination facility (e.g., sprout production units), adding Se to the soaking water is technically simple and low-cost. For open-field chestnut production, applying Se to soil or as a foliar spray is more common, but our study does not directly test those methods. The optimal concentration for field application would likely differ due to soil adsorption, rainfall, and microbial transformation. This study provides proof-of-concept for Se biofortification of germinated chestnuts under controlled conditions, but field application should be preceded by dose-optimization and safety trials.

## 3. Materials and Methods

### 3.1. Chemicals and Reagents

Sodium selenite was purchased from MedChem Express (Taian Havay Chemicals Co., Ltd., Taian, China). Se^IV^ and Se^VI^ standards were obtained from the National Institute of Metrology (Beijing, China). SeCys_2_, SeMet, and MeSeCys (methylselenocysteine) standards were purchased from Sigma-Aldrich (St. Louis, MO, USA). Protease XIV, lipase, Tris-HCl, L-phenylalanine, sodium borate, methionine, NBT, EDTA-Na_2_, riboflavin, potassium phosphate monobasic/dibasic, GSH, sodium azide, hydrogen peroxide (H_2_O_2_), Folin–Ciocalteu reagent, gallic acid, catechin, DPPH (2,2-diphenyl-1-picrylhydrazyl), ABTS (2,2′-azino-bis(3-ethylbenzothiazoline-6- sulfonic acid)), and Trolox were purchased from Sigma-Aldrich (St. Louis, MO, USA), while 2,3-Diaminonaphthalene was purchased from Macklin Biochemical Co., Ltd. (Shanghai, China). Potassium persulfate was purchased from Aladdin Biochemical Technology Co., Ltd. (Shanghai, China). Phosphate-buffered saline (PBS, 50 mmol/L, pH 7.8), EDTA, PVP-K30, Triton X-100, BCA protein assay kit, PAL activity assay kit, GPX activity assay kit, SOD activity assay kit, and CAT activity assay kit were purchased from Solarbio Science & Technology Co., Ltd. (Beijing, China). AccQ·Tag Ultraborate buffer and AccQ·Tag reagent were purchased from Waters Corporation (Milford, MA, USA).

### 3.2. Plant Materials and Treatments

The seeds of chestnut (*Castanea mollissima*) variety “Zaofeng” were harvested from healthy trees in the primary chestnut production region of Qinglong County, Qinhuangdao City, Hebei Province, China. The seeds were stored at −3 °C for preservation. Following the selection of uniform seeds, they were washed with sterile water, soaked for 12 h, and then evenly distributed on trays (50 cm × 30 cm × 15 cm) filled with sterilized sand mixed with sterile water (control) or varying concentrations (20, 40, 60, and 80 mg/L) of Na_2_SeO_3_. The trays were maintained at 22 °C and 85% relative humidity for 8 days [26]. Radicle emergence was observed on day 3, and full germination (radicle > 2 mm) was reached by day 4. Ten chestnut seeds per replicate were collected at 0, 2, 4, 6 and 8 days post-sowing, and the whole chestnuts were gently rinsed three times with deionized water to remove surface-adsorbed Se. The outer hard shell and the brown seed coat were then removed, and only the edible kernel was ground into powder after seed coat removal and stored at −40 °C for subsequent physiological and biochemical analysis. All results are expressed on a dry weight basis (dw) after drying a subsample at 105 °C to constant weight. Each treatment was replicated three times, with each replicate consisting of 30 seeds.

### 3.3. Determination of Se Content

#### 3.3.1. Total Se Content

The total selenium content was determined using fluorescence spectrophotometry (Cary Eclipse; Agilent Instruments, Santa Clara, CA, USA) [13]. The procedure was as follows: 0.5 g of dried chestnut powder was digested overnight by heating with 10 mL of a mixed solution of HNO_3_ and HClO_4_ (9:1, *v*/*v*). Subsequently, 5 mL of HCl (6 mol/L) was added to the digest. The heat-digested sample was then reacted with 2,3-diaminonaphthalene, and after extraction with cyclohexane, the fluorescence intensity was measured. A calibration curve was prepared using a selenium standard solution, and the total selenium content was calculated and expressed as mg/kg DW.

#### 3.3.2. Five Se Speciation Content

Accurately weigh 0.3000 g of dried germinated chestnut sample and place in a digestion vessel. Add 5 mL of HNO_3_ and 1 mL of H_2_O_2_, and perform microwave-assisted digestion. After digestion, cool the solution and transfer the digest to a 10 mL volumetric flask. Rinse the digestion vessel three times with ultrapure water, combine the washings, and dilute to the mark with ultrapure water. Subsequently, transfer 0.5 mL of the digest to a 20 mL centrifuge tube, and sequentially add 60 mg of protease XIV, 30 mg of lipase, and 10 mL of Tris-HCl buffer. Mix thoroughly, then ultrasonicate for 30 min. Place the tube in a constant temperature water bath shaker and shake horizontally at 37 °C and 150 r/min. After incubation in the dark for 20 h, centrifuge the sample at 10,000 r/min for 30 min. Filter the supernatant through a 0.22 μm aqueous filter membrane and store at −20 °C. Add 5 mL of hydrolysis solution containing 100 mg of catabolic enzyme to the remaining residue, and repeat the above steps. Combine the two supernatants and filter through a 0.22 μm aqueous filter membrane. Perform Se speciation analysis using HPLC-ICP-MS [65]. Prepare a mixed standard solution using Secys_2_ (>99% purity), MeSeCys (>99% purity), SeMet (>99% purity), Se^IV^ and Se^VI^ standards (>99% purity). Using the external standard method, construct a calibration curve by plotting Se concentration on the X-axis against chromatographic peak area on the Y-axis with a series of mixed standard solutions. The speciation content of Se in the sample was determined using the provided formula (1):

(1) $$X=\frac{(C\;{-\;C}_{0})\times V}{m}\times 1000$$

where: X—speciation content of Se in the sample (mg/kg); C—determination of concentration of sample extract (μg/L); C_0_—concentration of sample blank determination (μg/L); V—total volume of sample extract at constant volume (L); m—weight of sample (g).

#### 3.3.3. Calculation of Selenium Enrichment Rate

The enrichment rate of selenium was calculated by Equations (2) and (3), respectively [66].

O_se_ = C_SeCys2_ + C_MeSeCys_ + C_SeMet_ (2)

R_se_ (%) = O_se_/T_se_ × 100 (3)

where C_SeCys2_ represents the SeCys_2_ content of germinated chestnuts, C_MeSeCys_ represents the MeSeCys content of germinated chestnuts, C_SeMet_ represents the SeMet content of germinated chestnuts, T_se_ represents the total selenium content of germinated chestnuts, O_se_ represents the organic selenium content, and R_se_ represents the selenium enrichment rate.

### 3.4. Determination of PAL, GPX, SOD and CAT Activity

The extraction and activity assays of PAL, GPX, SOD, and CAT were performed following Du et al. (2024) [67]. Fresh germinated chestnut samples (3 g) were homogenized with 6 mL of ice-cold PBS (50 mmol/L, pH 7.8) containing 1 mmol/L EDTA, 2% (*m*/*v*) PVP-K30, and 0.3% Triton X-100. After grinding for 60 s, the mixture was centrifuged at 10,000× *g* for 5 min at 4 °C. The resulting supernatant served as the crude enzyme extract for all assays, with activity determination based on Zhang et al. (2025) [68].

PAL activity assay: A total of 0.1 mL of crude enzyme extract was mixed with 2.0 mL of 0.1 mol/L borate buffer (pH 8.8) and 1.0 mL of 0.02 mol/L L-phenylalanine solution. After thorough mixing, the mixture was incubated in a constant temperature water bath at 37 °C for 30 min. The reaction was terminated by adding 0.5 mL of 35% trichloroacetic acid (TCA). The absorbance was measured at 290 nm. One unit (U) of PAL activity was defined as the amount of enzyme causing an increase of 0.01 in absorbance at 290 nm per 30 min under the assay conditions.

SOD activity assay: A total of 0.1 mL of crude enzyme extract was sequentially mixed with 1.7 mL of PBS (50 mmol/L, pH 7.8), 0.3 mL of methionine (130 mmol/L), 0.3 mL of NBT (750 μmol/L), 0.3 mL of EDTA-Na_2_ (100 μmol/L), and 0.3 mL of riboflavin (20 μmol/L). A control tube was prepared by replacing the crude enzyme extract with an equal volume of PBS. The reaction mixture was illuminated under a fluorescent lamp (4000 lx) for 15 min, and then the absorbance was measured at 560 nm. One unit of SOD activity was defined as the amount of enzyme required to inhibit 50% of NBT photoreduction.

GPX activity assay: A total of 0.1 mL of crude enzyme extract was mixed with 0.6 mL of 50 mmol/L potassium phosphate buffer (pH 7.0), 0.1 mL of 1 mmol/L GSH, and 0.1 mL of 1 mmol/L sodium azide. After pre-incubation at 37 °C for 5 min, the reaction was initiated by adding 0.1 mL of H_2_O_2_ solution (1.5 mmol/L). The mixture was immediately vortexed and allowed to react for exactly 10 min. The reaction was terminated by adding 0.5 mL of 10% (*w*/*v*) TCA, followed by centrifugation at 10,000× *g* for 5 min at 4 °C. Then, 0.4 mL of the supernatant was mixed with 2.6 mL of 50 mmol/L potassium phosphate buffer (pH 7.0) and 0.5 mL of DTNB chromogenic solution (10 mmol/L). After incubation at room temperature for 5 min, the absorbance was measured at 412 nm. The control tube was prepared by replacing the crude enzyme extract with an equal volume of potassium phosphate buffer. One unit of GPX activity was defined as a change of 0.005 in absorbance at 412 nm per minute.

CAT activity assay: A total of 0.1 mL of crude enzyme extract was mixed with 2.9 mL of PBS (50 mmol/L, pH 7.0) containing 20 mmol/L H_2_O_2_. The activity was determined by monitoring the decrease in absorbance at 240 nm. The control solution was PBS with H_2_O_2_ but without enzyme. One unit of CAT activity was defined as a decrease of 0.01 in absorbance at 240 nm per minute.

All absorbance values were measured by a UV-Vis spectrophotometer (UT-1901; METASH, Shanghai, China), and all enzyme activities are expressed as U/g.

### 3.5. Determination of GABA Content

The procedures were based on Jannoey, P., et al. [69] with slight modifications. Briefly, 0.2 g of dried germinated chestnut sample power was extracted using 1 mL of 0.1 mol/L HCl at 70 °C for 1 h, and centrifuged at 12,000 rpm for 10 min. Then, 10 µL of the supernatant was mixed with 70 µL of AccQ·Tag Ultra Borate Buffer and 20 µL of accqtag reagent in a derivatization tube. After vortexing, the mixture was heated at 55 °C for 10 min and then cooled. HPLC analysis was performed at a flow rate of 0.5 mL/min, column temperature of 55 °C, and the injection volume was 1 μL. GABA content was quantified using a standard curve and expressed as mg/kg DW.

### 3.6. Determination of Total Polyphenol and Flavonoid Content

The extraction process for total polyphenol content (TPC) and total flavonoid content (TFC) was similar to that of the GABA. TPC was determined using the Folin–Ciocalteu method [66]. Briefly, 500 µL of sample extract was mixed with 250 µL of Folin–Ciocalteu reagent and 3.00 mL of distilled water. After 5 min, 750 µL of 7% Na_2_CO_3_ was added, and the mixture was incubated at 20 °C for 1 h. Absorbance was measured at 765 nm. TPC was quantified against a gallic acid calibration curve and expressed as mg gallic acid equivalents (GAE)/g DW. TFC was measured according to Ren et al. (2024) [70]. Briefly, 250 µL of sample extract was mixed with 1250 µL of distilled water and 75 µL of 5% NaNO_2_ solution. After 5 min, 150 µL of 10% AlCl_3_·6H_2_O was added; then, 500 µL of 1 mol/L NaOH and 275 µL of distilled water. TFC was calculated using a calibration using a catechin (CAE) standard curve and expressed as mg CAE/g DW. The absorbance of the mixture was measured at 510 nm. All absorbance values were measured by a UV-Vis spectrophotometer (UT-1901; METASH, Shanghai, China).

### 3.7. Determination of DPPH Radical Scavenging Activity

DPPH radical scavenging activity was measured using a modified method of Farhadi et al. (2016) [71]. In brief, 0.2 g of dried germinated chestnut sample power was extracted using 2 mL of methanol. After ultrasonic extraction at 100–200 W for 20 min, the mixture was centrifuged at 10,000× *g* for 5 min. Then, 100 µL of the supernatant was mixed with 3.9 mL of 0.1 mmol/L DPPH in methanol and kept in the dark for 30 min. Absorbance was then read at 517 nm using a UV-Vis spectrophotometer (UT-1901; METASH, Shanghai, China). The scavenging activity was quantified against a Trolox standard curve and expressed as mg Trolox equivalents per gram of dry weight (mg TE/g DW).

### 3.8. Determination of ABTS Radical Scavenging Activity

ABTS radical cation scavenging activity was determined according to the method of Spiegel et al. (2022) [72] with minor modifications. Briefly, 0.2 g of dried germinated chestnut sample power was extracted using 2 mL of methanol. After ultrasonic extraction at 100–200 W for 20 min, the mixture was centrifuged at 10,000× *g* for 5 min. Then, 100 µL of the supernatant was mixed with 1.9 mL of 0.325 mol/L phosphate buffer and 2.0 mL of ABTS working solution and incubated in the dark at room temperature for 10 min. The absorbance was measured at 734 nm using a UV-Vis spectrophotometer (UT-1901; METASH, Shanghai, China). A control was prepared by replacing the sample extract with the corresponding solvent. The scavenging activity was calculated as the percentage of inhibition and expressed as mg Trolox equivalents per gram of dry weight (mg TE/g DW) using a Trolox standard curve.

### 3.9. Statistical Analysis

All experiments were performed with three biological replicates, and the results are reported as mean values with standard errors. All statistical analyses were carried out using SPSS 22.0 (SPSS Inc. Chicago, IL, USA), while graphical analysis was conducted using Origin 2023 (Microcal Software, Northampton, MA, USA). One-way ANOVA followed by Tukey’s HSD post-hoc test (*p* < 0.05) was used to compare treatments.

## 4. Conclusions

In summary, exogenous Na_2_SeO_3_ treatment exerts dose-dependent effects on selenium biofortification, phenolic metabolism, antioxidant enzyme activities, and total antioxidant capacity in germinating chestnuts. Low selenium concentrations (20–40 mg/L) significantly promoted total selenium accumulation and the conversion of organic selenium (SeMet, SeCys_2_ and MeSecys), with the highest proportion of organic selenium (81.3%) observed at 20 mg/L. Concurrently, low selenium treatment upregulated the activities of PAL, GPX, SOD, and CAT, promoted the accumulation of γ-aminobutyric acid (GABA), total polyphenols and flavonoids, and consequently enhanced DPPH and ABTS radical scavenging activities. The 40 mg/L Na_2_SeO_3_ treatment was identified as the optimal concentration, yielding the greatest improvements across all measured parameters. In contrast, high selenium concentration (80 mg/L) gradually inhibited these beneficial effects, leading to a decreased proportion of organic selenium, reduced enzyme activities, diminished accumulation of phenolics and GABA, and weakened antioxidant capacity, indicating oxidative stress and metabolic disorder. These findings reveal that the functional activity of germinating chestnuts exhibits a “low-dose promotion, high-dose inhibition” response to selenium treatment, and establish 40 mg/L Na_2_SeO_3_ as the optimal concentration for producing selenium-enriched germinated chestnuts with improved nutritional quality and antioxidant potential. This study provides a theoretical basis and technical parameters for the development of functional foods from selenium-enriched germinated chestnuts.

## Data Availability

The original contributions presented in this study are included in the article; further inquiries can be directed to the corresponding author.
